# Time Trends and Monthly Variation in Swedish Acute Stroke Care

**DOI:** 10.3389/fneur.2019.01177

**Published:** 2019-11-07

**Authors:** David Darehed, Mathias Blom, Eva-Lotta Glader, Johan Niklasson, Bo Norrving, Marie Eriksson

**Affiliations:** ^1^Sunderby Research Unit, Department of Public Health and Clinical Medicine, Umeå University, Umeå, Sweden; ^2^Department of Clinical Sciences Lund, Medicine, Lund University, Lund, Sweden; ^3^Department of Public Health and Clinical Medicine, Umeå University, Umeå, Sweden; ^4^Sunderby Research Unit, Department of Community Medicine and Rehabilitation, Geriatric Medicine, Umeå University, Umeå, Sweden; ^5^Department of Clinical Sciences, Neurology, Lund University, Lund, Sweden; ^6^Department of Statistics, Umeå School of Business, Economics and Statistics, Umeå University, Umeå, Sweden

**Keywords:** stroke, quality of care, monthly variation, longitudinal trends, survival

## Abstract

**Background and Purpose:** Studies of monthly variation in acute stroke care have led to conflicting results. Our objective was to study monthly variation and longitudinal trends in quality of care and patient survival following acute stroke.

**Methods:** Our nationwide study included all adult patients (≥18 years) with acute stroke (ischemic or hemorrhagic), admitted to Swedish hospitals from 2011 to 2016, and that were registered in The Swedish Stroke Register (Riksstroke). We studied how month of admission and longitudinal trends affected acute stroke care and survival. We also studied resilience to this variation among hospitals with different levels of specialization.

**Results:** We included 132,744 stroke admissions. The 90-day survival was highest in May and lowest in January (84.1 vs. 81.5%). Thrombolysis rates and door-to-needle time within 30 min increased from 2011 to 2016 (respectively, 7.3 vs. 12.8% and 7.7 vs. 28.7%). Admission to a stroke unit as first destination of hospital care was lowest in January and highest in June (78.3 vs. 80.5%). Stroke unit admission rates decreased in university hospitals from 2011 to 2016 (83.4 vs. 73.9%), while no such trend were observed in less specialized hospitals. All the differences above remained significant (*p* < 0.05) after adjustment for possible confounding factors.

**Conclusion:** We found that month of admission and longitudinal trends both affect quality of care and survival of stroke patients in Sweden, and that the effects differ between hospital types. The observed variation suggests an opportunity to improve stroke care in Sweden. Future studies ought to focus on identifying the specific factors driving this variation, for subsequent targeting by quality improvement efforts.

## Introduction

Studies of monthly variation in acute stroke care show lower mortality during the summer and early autumn in the Northern hemisphere and in March in the Southern hemisphere (1–3). This variation seems to be mostly driven by seasonality in cardiovascular, cerebrovascular and respiratory diseases, while other groups of patients, such as those suffering from lung cancer or those dying from accidents, show no clear seasonality (4). For stroke patients the currently published results are conflicting, with some studies showing no seasonal variation and others showing a higher mortality rate during winter (5–8). From Sweden, only one single-center study of seasonal variation found higher mortality rates during winter (9).

Apart from a general seasonal variation, some of the previous studies have addressed the so-called “July effect,” which suggests that quality-of-care decreases during trainee changeovers at the end of the academic year (10, 11). A systematic review that summarized these studies found some evidence that mortality and efficiency of care had a tendency to worsen at the time of academic year-end changeovers (12). However, the studies are largely heterogeneous, and analysis of possible contributing causes are lacking (12, 13). We believe that identifying variations in quality of care resulting from academic year-end changeovers and other organizational factors is paramount, in that such factors can be targeted with quality improvement efforts that may translate into lives saved.

Stroke is the second leading cause of death worldwide, although incidence rates are decreasing globally (14). Swedish data reflect this trend and show a decline in stroke incidence over time (15). Stroke care seems also to be dynamic over time with, for instance, increasing rates of thrombolysis and decreased door-to-needle (DTN) times in Sweden over the past several years (15). We believe that such long-term changes may reflect the increased use, over time, of care management algorithms (such as thrombolytic therapy or catheter-based interventions) since their introduction.

We hypothesized that there are monthly variations and longitudinal time trends in the quality of stroke care in Sweden, and that these variations manifest themselves as differences in overall survival as well as in other quality-of-care measures. While quantifying these trends was the main objective of the study, we also chose to explore whether these trends varied in magnitude among hospitals with different levels of specialization.

## Materials and Methods

### Setting

Sweden is a country in Northern Europe with 10 million inhabitants. Twenty-one different regions and counties provide healthcare in Sweden, and healthcare is mostly tax-funded apart from a minor copayment made by the patient. Overall, 72 hospitals (nine university hospitals, 22 specialized non-university hospitals, and 41 community hospitals) care for all acute stroke patients.

### Study Design and Patients

We conducted this retrospective register-based cohort study on data from The Swedish Stroke Register (Riksstroke), a nationwide quality register. All 72 hospitals that care for stroke patients are included in Riksstroke (15). The register holds information on acute care, patient reported outcomes at 3 and 12 months, as well as all-cause mortality data. Variables include temporal measurements, acute and secondary prevention, patient characteristics and health-related outcomes. The register has an estimated nationwide coverage of around 95% of all acute stroke patients admitted to hospital (16, 17).

We included all adult patients (≥18 years) admitted to any of the 72 hospitals, that were registered with an acute hemorrhagic or ischemic stroke (ICD-10 codes I61 and I63) between 2011 and 2016. We excluded patients with transient ischemic attack and patients already hospitalized. Mortality data were complete from January 2011 to April 2017, which allowed for measuring survival at both 7 and 90 days during the entire study period. Thrombectomy was not studied as an outcome since most of the study period includes the time before the positive results of thrombectomy studies. All patients are informed about registration in Riksstroke, and are informed of their rights to decline participation (opt-out consent). The regional ethics review board in Umeå, Sweden, approved the study (DNR 2016/346-31). Because of the sensitive nature of the data collected for this study, requests to access the data set from qualified researchers trained in human subject confidentiality protocols may be sent to Professor Bo Norrving at Riksstroke.

### Statistical Methods and Variables

Main exposure variables were month of admission (recorded as January, February, …until December), year of admission (2011, 2012, …until 2016) and longitudinal months (January 2011, February 2011, …until December 2016). Our target variables included quality-of-care indicators (thrombolysis; DTN time within 30 min; DTN time within 60 min; admission to a stroke unit as first destination of hospital care; swallowing test; physiotherapist assessment within 48 h; and occupational therapist assessment within 48 h) and survival at 7 and 90 days.

Descriptive analyses of patient characteristics, missing data, and quality of care were followed by multivariable logistic regression analyses of the quality-of-care indicators and survival in separate models. We included the following variables in the models: age (in years, 18–64, 65–74, 75–84, and 85+), stroke type (hemorrhagic, ischemic), sex, activities of daily living dependency (the need of help with toiletry and/or clothing) before admission, co-morbidity (previous stroke diagnosis, atrial fibrillation, diabetes mellitus of any type, hypertensive treatment), hospital (all 72 hospitals), level of consciousness upon arrival [using the reaction level scale [RLS]: RLS 1 [awake], RLS 2–3 [drowsy], RLS 4–8 [unconscious]], arrival by ambulance, and smoking. The degree of missing data was low, except for the two variables “arrival by ambulance” (6.6%) and “smoking” (9.7%). We handled missing data for these variables by including them in separate categories. A validation of Riksstrokes assessment of ADL have shown good agreement with Barthel's index (18). Analyses of thrombolysis were restricted to patients with ischemic stroke, and analyses of DTN times were restricted to patients receiving thrombolysis within 9 h from hospital admission. “January” and “2011” were chosen as reference categories in all adjusted analyses. We chose our covariates based on clinical expertise and availability in Riksstroke.

We studied hospital resilience to monthly variation and longitudinal trends in quality of care by including interaction terms between month and year of admission, and the hospital's level of specialization. Reference categories in the interaction terms were “January,” “2011,” and “specialized non-university hospitals.” Only variables with a significant interaction term (*p* < 0.05) were then included in unadjusted and adjusted stratified analyses for each hospital type. All statistical analyses were conducted using IBM® SPSS® Statistics 24.

## Results

### Patient Characteristics

A total of 132,744 patient admissions matched the inclusion criteria and were included in the analyses. Of these patients, 52% were men, 62% had hypertensive treatment and the mean age was 75 years. Ischemic strokes accounted for 87% of all admissions, and 84% of the patients were alert upon presentation. In total, 20% of the admissions were to university hospitals, 46% to specialized non-university hospitals and 34% to community hospitals. The number of annual stroke admissions decreased over time from 23,334 in 2011 to 20,277 in 2016 (Table 1 and Table SI in the Supplementary Material).

**Table 1:** Patient characteristics.

	**Total *N* (%)**	**Missing *N* (%)**
Total	132,744 (100%)	–
Sex:	–	0
Men	69,625 (52.5%)	–
Women	63,119 (47.5%)	–
Age (mean/median)	75.5/77	0
ADL^*^ dependency	15,710 (12.2%)	3,995 (3.0%)
Previous stroke	31,236 (23.7%)	716 (0.5%)
Atrial fibrillation	37,722 (28.6%)	622 (0.5%)
Diabetes	27,687 (20.9%)	470 (0.4%)
Hypertensive treatment	81,656 (61.9%)	805 (0.6%)
Smoking	17,006 (14.2%)	12,890 (9.7%)
Arrival by ambulance	94,052 (75.9%)	8,817 (6.6%)
Consciousness:	–	1,450 (1.1%)
Alert	109,792 (83.6%)	–
Drowsy	15,117 (11.5%)	–
Unconscious	6,385 (4.9%)	–
Stroke type:	–	0
Hemorrhagic	17,037 (12.8%)	–
Ischemic	115,707 (87.2%)	–
Hospital type	–	0
Specialized non-university	60,458 (45.5%)	–
University	27,126 (20.4%)	–
Community	45,160 (34.0%)	–

* *ADL, activities of daily living*.

Patient characteristics did not vary substantially during the study period (Tables SSI, SII in the Supplementary Material). Complete information on patient characteristics and missing cases is reported in Table 1 and Tables SI–SV in the Supplementary Material.

### Survival

**Figure 1:**
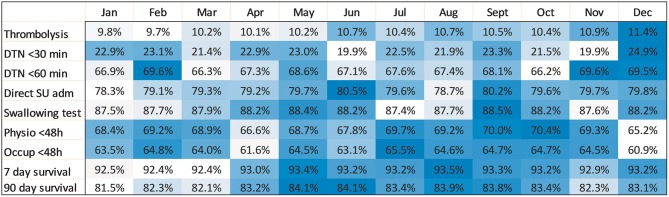
Heatmap with unadjusted analyses of survival and all quality-of-care indicators per month. Darker blue color indicates higher rates.

**Figure 2:**
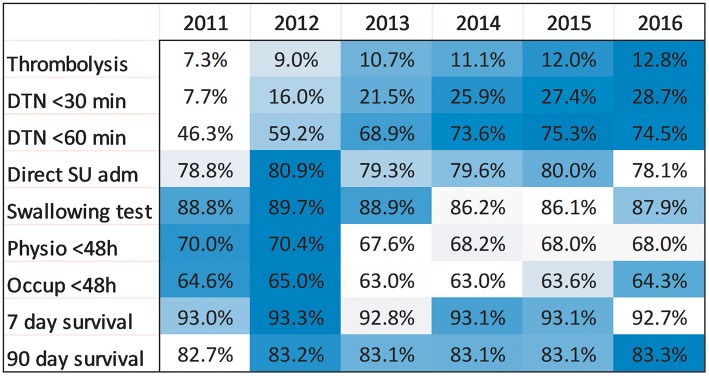
Heatmap with unadjusted analyses of survival and all quality-of-care indicators per year. Darker blue color indicates higher rates.

**Figure 3:**
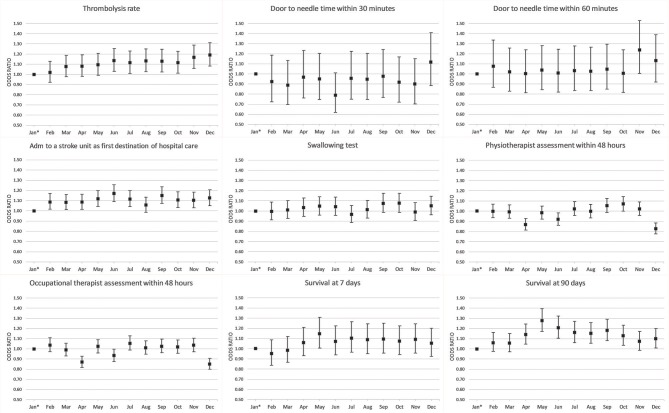
Adjusted analyses of quality-of-care indicators and survival per month. *Reference.

**Figure 4:**
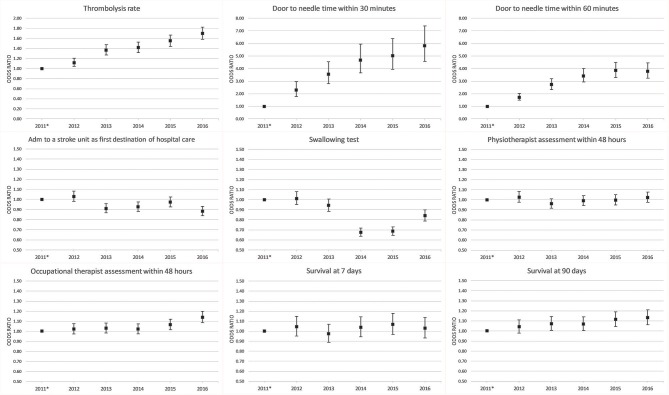
Adjusted analyses of quality-of-care indicators and survival per year. *Reference.

The 90-day survival showed a monthly variation, with the lowest rate recorded in January (81.5%) and the highest rate in May (84.1%) (adjusted OR, 1.28; 95% CI, 1.17–1.40). Ninety-day survival also slightly increased from 82.7% in 2011 to 83.3% in 2016 (adjusted OR, 1.13; 95% CI, 1.06–1.21). Seven-day survival also showed a monthly variation with the lowest rate recorded in February (92.4%) and the highest rate in August (93.5%) (adjusted OR, 1.14; 95% CI, 1.00–1.31). From 2011 to 2016, 7 day survival decreased from 93.0 to 92.7% (adjusted OR, 1.03; 95% CI, 0.93–1.14) (Figures 1–4). There were no significant differences in survival between hospital types (Table 2).

**Table 2:** Significance test for interaction terms between hospital type by month of admission, and hospital type by year of admission.

**Hospital type by month**	***P*-value**	**Hospital type by year**	***P*-value**
Thrombolysis	0.880	Thrombolysis	**0.043**
DTN <30 min	0.807	DTN <30 min	**0.000**
DTN <60 min	0.647	DTN <60 min	**0.027**
Stroke unit admission	**0.000**	Stroke unit admission	**0.000**
Swallowing test	**0.003**	Swallowing test	**0.000**
Physio <48 h	0.066	Physio <48 h	**0.000**
Occupational <48 h	0.209	Occupational <48 h	**0.000**
Survival at 7 days	0.742	Survival at 7 days	0.223
Survival at 90 days	0.782	Survival at 90 days	0.168

*Bold numbers are significant outcomes (p < 0.05)*.

### Stroke Care

**Figure 5:**
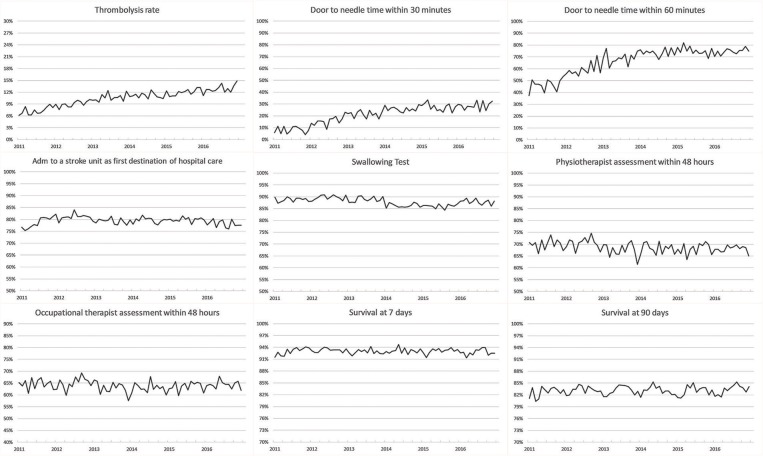
Unadjusted monthly longitudinal analyses.

**Figure 6:**
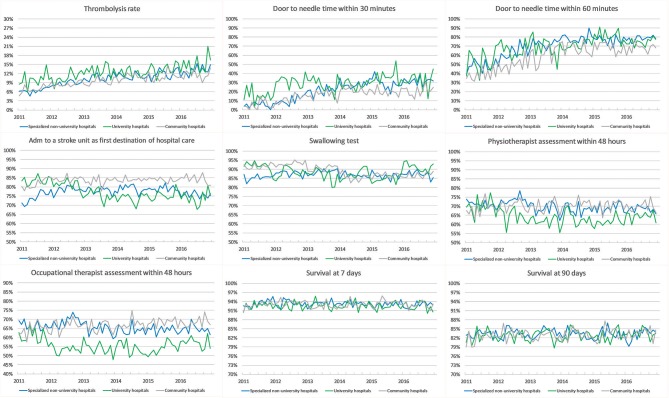
Unadjusted monthly longitudinal analyses stratified by hospital type.

**Figure 7:**
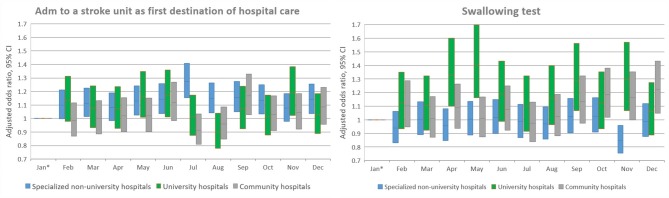
Adjusted analyses per month stratified by hospital type. OR with 95% confidence intervals for each hospital type. Only outcomes with significant interaction terms are included. *Reference.

**Figure 8:**
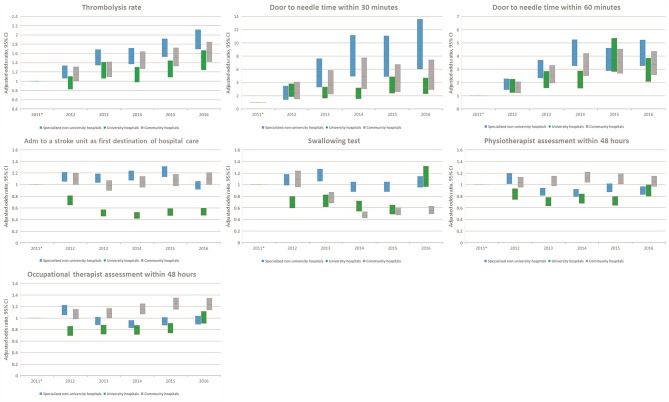
Adjusted analyses per year stratified by hospital type. OR with 95% confidence intervals for each hospital type. Only outcomes with significant interaction terms are included. *Reference.

Thrombolysis rates increased continuously from 7.3% in 2011 to 12.8% in 2016 (adjusted OR, 1.70; 95% CI, 1.58–1.83). The increased rates during the study period were also reflected by an increased monthly rate from 9.8% in January to 11.4% in December (adjusted OR, 1.19; 95% CI, 1.08–1.31). From 2011 to 2016, university hospitals had the highest thrombolysis rates (10.2–14.7%) while community hospitals had the lowest rates (6.9–11.6%). Specialized non-university hospitals had the highest increase in thrombolysis rates (from 6.4 to 12.9%), which was significantly higher than that of university hospitals (respectively, OR, 1.89; 95% CI, 1.69–2.11; and OR, 1.44; 95% CI, 1.24–1.67) (Table 2 and Figures 1–8).

DTN times within 30 min increased from 7.7% in 2011 to 28.7% in 2016 (adjusted OR, 5.81; 95% CI, 4.57–7.39), while DTN times within 60 min increased from 46.3% in 2011 to 74.5% in 2016 (adjusted OR, 3.80; 95% CI, 3.24–4.44). DTN times did not differ significantly among months, except for November, when the DTN time within 60 min was slightly more frequent (OR, 1.24; 95% CI, 1.00–1.53). DTN times within 30 min showed a dip in June, although this difference was not significant (OR, 0.79; 95% CI, 0.62–1.01). Community hospitals had the lowest rates of DTN times both within 30 and 60 min. University hospitals started with higher rates than those of community and specialized non-university hospitals. However, by the end of the study, the rates were at similar levels since the rates at specialized non-university hospitals increased significantly more, especially for DTN within 30 min (from 5.2% in 2011 to 31.6% in 2016) (Table 2 and Figures 1–8).

Admission to a stroke unit as first destination of hospital care decreased from 78.8% in 2011 to 78.1% in 2016 (adjusted OR, 0.88; 95% CI, 0.84–0.93). This change was driven by university hospitals, where the rate decreased from 83.4 to 73.9% (adjusted OR, 0.53; 95% CI, 0.47–0.59), while community and specialized non-university hospitals slightly increased their admission rates from 2011 to 2016 (respectively, 82.0–84.2%, and 74.4–75.7%). When comparing admission rates among different months, January displayed the lowest rates (78.3%) and June the highest ones (80.5%) (Table 2 and Figures 1–8).

We observed small fluctuations in swallowing test rates, with the lowest monthly rate in July (87.4%) and the highest in September (88.5%); also, the lowest yearly rate was recorded in 2015 (86.1%) and the highest in 2012 (89.7%). Physiotherapist assessment rates within 48 h were lowest in December (65.2%) and highest in October (70.4%). Occupational therapist assessment rates within 48 h were of 60.9% in December and 65.5% in July. The assessment rates were relatively similar among the years (Table 2 and Figures 1, 2, 4–8).

## Discussion

Our study confirms that acute stroke care in Sweden displays significant monthly variation as well as longitudinal time trends, and that the magnitude of the variation differs across quality-of-care indicators. We also found that hospitals with various levels of specialization are differentially resilient to this variation.

The 7-day survival was highest in August and the 90-day survival was highest in May. This is in line with the general mortality patterns in Sweden and other countries in the Northern hemisphere. In these areas, most deaths occur during winter and fewer occur during summer and autumn, contrary to the hypothesis of the July effect (1–3). Seven-day survival rates showed a smaller monthly variation and peaked later in the year, compared to 90-day survival. This shows that the impact of the monthly variation increases with longer follow up time. We believe that the reasons for these effects are multifactorial, and one such factor could be seasonality in infectious diseases where for example the flu peaks between December to April in Europe (19). Given our findings, we suggest that monthly variation should be considered as a confounding factor in studies of stroke mortality. When comparing survival between hospitals, a study from the US found that major teaching hospitals had lower mortality rates than non-teaching hospitals for patients with common conditions (20). In our analyses, we found no evidence that different hospital types were differentially susceptible to the monthly variation in terms of survival.

Thrombolysis and DTN times are considered good indicators of quality of care in the most acute phase of the stroke care chain. We believe that the increased thrombolysis rates over time indicate the increased use of this procedure. This observation may be explained by the effective information and organizational feedback related to the use of Riksstroke, having the goal of reaching thrombolysis rates of 15% during the study period. One of the strengths of using a national stroke quality register is that it not only monitors the quality of care but can also actively give feedback to healthcare providers allowing them to adjust. Another factor that likely had an impact on thrombolysis rates is that the Swedish National Board of Health and Welfare updated their recommendations on thrombolysis in 2014, making also patients aged 80 years and older eligible for thrombolysis (21). The improvement in DTN times also likely reflects the maturation of the thrombolysis procedure in Sweden over time, potentially influenced by the systematic reporting and feedback from Riksstroke. When we compared hospital types, all had better results over time. Specifically, university hospitals displayed better results already at the start of the study period, whereas specialized non-university and community hospitals were lagging a few years behind but eventually caught up with university hospitals. This probably reflects the fact that new knowledge first is implemented in higher competence centers, and then eventually spreads to smaller centers.

Admission to a stroke unit as first destination of hospital care is, as we see it, a strictly organizational factor. Previous studies have shown that stroke unit care increases both survival and independence (22). Unfortunately, our study shows a decreased rate of stroke unit admissions over time, where university hospitals alone accounted for this decrease. We believe the reason for this is a shortage of hospital beds, as shown in our previous work (23), since university hospitals have fewer stroke unit beds per capita than specialized non-university hospitals and community hospitals (24). The solution to this problem is likely multifactorial. We believe that mitigating bed shortages, as well as implementing local guidelines that prioritize stroke patients to stroke units, are some of the key factors for increasing the rates of admission to a stroke unit as first destination of hospital care.

Swallowing test rates, as well as physiotherapist and occupational therapist assessment rates, mostly reflect quality of care during an acute care episode. These three quality-of-care indicators did not significantly change over time, except for the occupational therapy assessment within 48 h that showed a minor increase. We believe the reason for this is the use of local guidelines prioritizing assessment, a parameter that is also reported as a quality indicator in Riksstroke. April, June, and December displayed the lowest assessment rates of the year; we believe that this reflects staffing practices, since physiotherapists and occupational therapists generally do not work during the Easter, Midsummer and Christmas holidays, hence increasing the time to assessment beyond 48 h. These differences should be possible to target by changing the staffing practices.

### Strengths and Limitations

Given that all Swedish hospitals caring for stroke patients report data to Riksstroke, along with a coverage of approximately 95% of stroke cases, we assess the risk of selection bias as low (15, 17). Riksstroke also undergoes extensive internal data validation, with no indications of systematic differences in data quality between hospitals (25). The rate of missing data was also low; this, along with the prospective nature of data collection in Riksstroke, vouches for better data quality than that obtained by using sources that accumulate data as a by-product of routine care delivery (i.e., electronic health records). Our results remained significant after adjusting for confounding variables, indicating that the findings are robust. Stroke severity measured as National Institutes of Health Stroke Scale (NIHSS) would be somewhat more granular than RLS. However, missing values of 50% for NIHSS made RLS the only viable choice. In addition to this, RLS has been proven in the past to be a good proxy in predicting stroke mortality (26).

## Conclusion

Our findings suggest that the quality of care and survival of acute stroke patients in Sweden displays a monthly variation as well as long-term trends. Most importantly, the magnitude of these effects differs between hospital types, suggesting an availability of best practices in some hospitals, which can be used to inspire quality improvement efforts among the laggards.

## Data Availability Statement

The datasets generated for this study will not be made publicly available because of the sensitive nature of the data collected for this study, requests to access the data set from qualified researchers trained in human subject confidentiality protocols may be sent to BN at Riksstroke.

## Ethics Statement

The studies involving human participants were reviewed and approved by the regional ethics review board in Umeå, Sweden DNR 2016/346-31. Written informed consent for participation was not required for this study in accordance with the national legislation and the institutional requirements.

## Author Contributions

All authors conceived, designed, and amended the study, discussed the ethical aspects of the study, and contributed to the subsequent and final drafts. DD and ME coordinated the study throughout and secured ethical approval from the ethics review board in Umeå. DD gathered, cleaned, and analyzed the data, and wrote the first draft of the manuscript.

### Conflict of Interest

The authors declare that the research was conducted in the absence of any commercial or financial relationships that could be construed as a potential conflict of interest.
