# Supplementary material for: Time Trends and Monthly Variation in Swedish Acute Stroke Care
Source: Front Neurol. 2019 Nov 7;10:1177. doi: 10.3389/fneur.2019.01177 (PMC6854029; doi:10.3389/fneur.2019.01177)
Supplement: Supplementary file 1 [file Table_1.DOCX]

Supplementary Material

Table I. Patient characteristics per year. n (%). *ADL = Activities of daily living. †RLS = Reaction level scale

|  | 2011 | 2012 | 2013 | 2014 | 2015 | 2016 |
| --- | --- | --- | --- | --- | --- | --- |
| Total admissions  Sex:  Men  Women | 23334 (17.6%)  12131 (52.0%)  11203 (48.0%) | 23207 (17.5%)  12008 (51.7%)  11199 (48.3%) | 22558 (17.0%)  11793 (52.3%)  10765 (47.7%) | 21992 (16.6%)  11562 (52.6%)  10430 (47.4%) | 21376 (16.1%)  11338 (53.0%)  10038 (47.0%) | 20277 (15.3%)  10793 (53.2%)  9484 (46.8%) |
| Age (mean/median) | 76/78 | 76/77 | 76/77 | 75/77 | 75/77 | 75/77 |
| ADL* dependency | 2651 (11.6%) | 2666 (11.7%) | 2659 (12.1%) | 2591 (12.1%) | 2673 (13.1%) | 2470 (12.8%) |
| Previous stroke | 5758 (24.9%) | 5675 (24.6%) | 5315 (23.7%) | 5094 (23.3%) | 4874 (22.9%) | 4520 (22.4%) |
| Atrial fibrillation | 6564 (28.4%) | 6406 (27.7%) | 6448 (28.8%) | 6428 (29.3%) | 6186 (29.0%) | 5690 (28.1%) |
| Diabetes | 4715 (20.3%) | 4775 (20.6%) | 4758 (21.2%) | 4659 (21.2%) | 4479 (21.0%) | 4301 (21.3%) |
| Hypertensive treatment | 14084 (60.8%) | 14150 (61.3%) | 14033 (62.7%) | 13690 (62.6%) | 13211 (62.1%) | 12488 (61.9%) |
| Smoking | 2958 (13.8%) | 3008 (14.0%) | 2859 (13.7%) | 2826 (14.2%) | 2723 (14.5%) | 2632 (15.1%) |
| Arrival by ambulance | 14271 (75.6%) | 16687 (75.9%) | 16583 (76.5%) | 16188 (76.3%) | 15712 (76.1%) | 14611 (74.8%) |
| Consciousness (RLS†)  Alert  Drowsy  Unconscious | 19094 (82.7%)  2767 (12.0%)  1231 (5.3%) | 19045 (83.0%)  2829 (12.3%)  1072 (4.7%) | 18527 (83.2%)  2616 (11.7%)  1122 (5.0%) | 18287 (84.0%)  2474 (11.4%)  1016 (4.7%) | 17832 (84.3%)  2332 (11.0%)  978 (4.6%) | 17007 (84.7%)  2099 (10.5%)  966 (4.8%) |
| Stroke type  Hemorrhagic  Ischemic | 2877 (12.3%)  20457 (87.7%) | 2834 (12.2%)  20373 (87.8%) | 2955 (13.1%)  19603 (86.9%) | 2844 (12.9%)  19148 (87.1%) | 2840 (13.3%)  18536 (86.7%) | 2687 (13.3%)  17590 (86.7%) |
| Hospital type  Specialized non-university  University  Community | 10623 (45.5%)  4772 (20.5%)  7939 (34.0%) | 10682 (46.0%)  4657 (20.1%)  7868 (33.9%) | 10180 (45.1%)  4662 (20.7%)  7716 (34.2%) | 9986 (45.4%)  4394 (20.0%)  7612 (34.6%) | 9647 (45.1%)  4435 (20.7%)  7294 (34.1%) | 9340 (46.1%)  4206 (20.7%)  6731 (33.2%) |

Table IIa. Patient characteristics per month from January to June. n (%). *ADL = Activities of daily living. †RLS = Reaction level scale

|  | January | February | March | April | May | June |
| --- | --- | --- | --- | --- | --- | --- |
| Total  Sex:  Men  Women | 11783 (8.9%)  6144 (52.1%)  5639 (47.9%) | 10586 (8.0%)  5418 (51.2%)  5168 (48.8%) | 11569 (8.7%)  6041 (52.2%)  5528 (47.8%) | 11363 (8.6%)  6024 (53.0%)  5339 (47.0%) | 11470 (8.6%)  5978 (52.1%)  5492 (47.9%) | 10756 (8.1%)  5745 (53.4%)  5011 (46.6%) |
| Age (mean/median) | 76/77 | 76/77 | 76/77 | 75/77 | 75/77 | 75/77 |
| ADL* dependency | 1451 (12.7%) | 1238 (12.1%) | 1404 (12.5%) | 1378 (12.5%) | 1418 (12.7%) | 1235 (11.8%) |
| Previous stroke | 2821 (24.1%) | 2533 (24.1%) | 2725 (23.7%) | 2741 (24.2%) | 2695 (23.6%) | 2485 (23.2%) |
| Atrial fibrillation | 3468 (29.6%) | 3012 (28.6%) | 3332 (28.9%) | 3236 (28.6%) | 3259 (28.6%) | 2991 (27.9%) |
| Diabetes | 2506 (21.3%) | 2245 (21.3%) | 2425 (21.0%) | 2355 (20.8%) | 2422 (21.2%) | 2219 (20.7%) |
| Hypertensive treatment | 7217 (61.6%) | 6528 (61.9%) | 7091 (61.6%) | 7007 (62.0%) | 7049 (61.8%) | 6582 (61.5%) |
| Smoking | 1496 (14.0%) | 1271 (13.3%) | 1414 (13.5%) | 1447 (14.1%) | 1497 (14.4%) | 1442 (14.9%) |
| Arrival by ambulance | 8456 (77.0%) | 7550 (76.5%) | 8216 (75.9%) | 8005 (75.5%) | 8085 (75.6%) | 7564 (75.4%) |
| Consciousness (RLS†)  Alert  Drowsy  Unconscious | 9641 (82.7%)  1439 (12.3%)  573 (4.9%) | 8737 (83.5%)  1192 (11.4%)  529 (5.1%) | 9465 (82.7%)  1375 (12.0%)  607 (5.3%) | 9405 (83.6%)  1304 (11.6%)  543 (4.8%) | 9536 (84.0%)  1272 (11.2%)  551 (4.9%) | 8958 (84.0%)  1190 (11.2%)  516 (4.8%) |
| Stroke type  Hemorrhagic  Ischemic | 10233 (86.8%)  1550 (13.2%) | 9225 (87.1%)  1361 (12.9%) | 10061 (87.0%)  1508 (13.0%) | 9885 (87.0%)  1478 (13.0%) | 10100 (88.1%)  1370 (11.9%) | 9380 (87.2%)  1376 (12.8%) |
| Hospital type  Specialized non-university  University  Community | 5390 (45.7%)  2447 (20.8%)  3946 (33.5%) | 4833 (45.7%)  2222 (21.0%)  3531 (33.4%) | 5272 (45.6%)  2408 (20.8%)  3889 (33.6%) | 5114 (45.0%)  2322 (20.4%)  3927 (34.6%) | 5104 (44.5%)  2344 (20.4%)  4022 (35.1%) | 4902 (45.6%)  2237 (20.8%)  3617 (33.6%) |

Table IIb. Patient characteristics per month from July to December. n (%). *ADL = Activities of daily living. †RLS = Reaction level scale

|  | July | August | September | October | November | December |
| --- | --- | --- | --- | --- | --- | --- |
| Total  Sex:  Men  Women | 10483 (7.9%)  5557 (53.0%)  4926 (47.0%) | 10905 (8.2%)  5774 (52.9%)  5131 (47.1%) | 10771 (8.1%)  5636 (52.3%)  5135 (47.7%) | 11179 (8.4%)  5877 (52.6%)  5302 (47.4%) | 10812 (8.1%)  5614 (51.9%)  5198 (48.1%) | 11067 (8.3%)  5817 (52.6%)  5250 (47.4%) |
| Age (mean/median) | 75/77 | 75/77 | 75/77 | 75/77 | 76/77 | 76/77 |
| ADL* dependency | 1262 (12.4%) | 1254 (11.9%) | 1234 (11.8%) | 1301 (12.0%) | 1274 (12.2%) | 1261 (11.8%) |
| Previous stroke | 2453 (23.5%) | 2595 (24.0%) | 2445 (22.8%) | 2642 (23.8%) | 2544 (23.7%) | 2557 (23.2%) |
| Atrial fibrillation | 2923 (28.0%) | 3012 (27.7%) | 3023 (28.2%) | 3117 (28.1%) | 3110 (29.0%) | 3239 (29.4%) |
| Diabetes | 2195 (21.0%) | 2275 (20.9%) | 2230 (20.8%) | 2283 (20.5%) | 2261 (21.0%) | 2271 (20.6%) |
| Hypertensive treatment | 6460 (62.1%) | 6688 (61.8%) | 6601 (61.6%) | 6843 (61.7%) | 6672 (62.2%) | 6918 (62.8%) |
| Smoking | 1377 (14.7%) | 1446 (14.7%) | 1416 (14.6%) | 1432 (14.2%) | 1418 (14.6%) | 1350 (13.5%) |
| Arrival by ambulance | 7338 (75.4%) | 7595 (75.1%) | 7554 (75.2%) | 7897 (75.3%) | 7762 (76.5%) | 8030 (77.0%) |
| Consciousness (RLS†)  Alert  Drowsy  Unconscious | 8658 (83.6%)  1174 (11.3%)  519 (5.0%) | 9100 (84.3%)  1216 (11.3%)  482 (4.5%) | 8936 (83.8%)  1218 (11.4%)  507 (4.8%) | 9241 (83.9%)  1265 (11.5%)  512 (4.6%) | 8906 (83.5%)  1216 (11.4%)  548 (5.1%) | 9209 (84.0%)  1256 (11.5%)  498 (4.5%) |
| Stroke type  Hemorrhagic  Ischemic | 9226 (88.0%)  1257 (12.0%) | 9504 (87.2%)  1401 (12.8%) | 9425 (87.5%)  1346 (12.5%) | 9736 (87.1%)  1443 (12.9%) | 9313 (86.1%)  1499 (13.9%) | 9619 (86.9%)  1448 (13.1%) |
| Hospital type  Specialized non-university  University  Community | 4777 (45.6%)  2108 (20.1%)  3598 (34.3%) | 5046 (46.3%)  2184 (20.0%)  3675 (33.7%) | 4877 (45.3%)  2184 (20.3%)  3710 (34.4%) | 5133 (45.9%)  2230 (19.9%)  3816 (34.1%) | 4938 (45.7%)  2126(19.7%)  3748 (34.7%) | 5072 (45.8%)  2314 (20.9%)  3681 (33.3%) |

Table III. Missing data on quality-of-care variables, survival, month and year of admission.

|  | Missing N/Total N | Missing % |
| --- | --- | --- |
| Thrombolysis | 188/115652 | 0.2% |
| Door-to-needle <30 min | 371/12019 | 3.1% |
| Door-to-needle <60 min | 371/12019 | 3.1% |
| Direct stroke unit admission | 852/132744 | 0.6% |
| Swallowing Test | 1/132744 | 0% |
| Occupational therapy assessment <48 h | 15711/132744 | 11.8% |
| Physiotherapist assessment <48 h | 15360/132744 | 11.6% |
| 7-day survival | 12/132744 | 0% |
| 90-day survival | 12/132744 | 0% |
| Month of admission | 0/132744 | 0% |
| Year of admission | 0/132744 | 0% |

Table IV. Missing data per year.

|  | 2011 | 2012 | 2013 | 2014 | 2015 | 2016 |
| --- | --- | --- | --- | --- | --- | --- |
| Thrombolysis | 27/20449 (0.1%) | 15/20363 (0.1%) | 75/19594 (0.4%) | 21/19141 (0.1%) | 30/18525 (0.2%) | 20/17580 (0.1%) |
| Door-to-needle <30 min | 85/1490 (5.7%) | 100/1838 (5.4%) | 75/2086 (3.6%) | 48/2129 (2.3%) | 31/2224 (1.4%) | 32/2252 (1.4%) |
| Door-to-needle <60 min | 85/1490 (5.7%) | 100/1838 (5.4%) | 75/2086 (3.6%) | 48/2129 (2.3%) | 31/2224 (1.4%) | 32/2252 (1.4%) |
| Direct stroke unit adm. | 50/23334 (0.2%) | 48/23207 (0.2%) | 131/22558 (0.6%) | 141/21992 (0.6%) | 284/21376 (1.3%) | 198/20277 (1.0%) |
| Swallowing test | 0/23334 (0.0%) | 0/23207 (0.0%) | 0/22558 (0.0%) | 1/21992 (0.0%) | 0/21376 (0.0%) | 0/20277 (0.0%) |
| Physiotherapy <48 h | 6106/23334 (26.2%) | 5901/23207 (25.4%) | 1088/22558 (4.8%) | 915/21992 (4.2%) | 790/21376 (3.7%) | 560/20277 (2.8%) |
| Occupational therapy <48 h | 6302/23334 (27.0%) | 5919/23207 (25.5%) | 1089/22558 (4.8%) | 933/21992 (4.2%) | 861/21376 (4.0%) | 607/20277 (3.0%) |
| 7-day survival | 4/23334 (0.0%) | 1/23207 (0.0%) | 1/22558 (0.0%) | 1/21992 (0.0%) | 4/21376 (0.0%) | 1/20277 (0.0%) |
| 90-day survival | 4/23334 (0.0%) | 1/23207 (0.0%) | 1/22558 (0.0%) | 1/21992 (0.0%) | 4/21376 (0.0%) | 1/20277 (0.0%) |

Table V. Missing data per month.

|  | January | February | March | April | May | June |
| --- | --- | --- | --- | --- | --- | --- |
| Thrombolysis | 6/10230 (0.1%) | 12/9223 (0.1%) | 20/10060 (0.2%) | 16/9883 (0.2%) | 4/10096 (0.0%) | 6/9369 (0.1%) |
| Door-to-needle <30 min | 33/1006 (3.3%) | 26/898 (2.9%) | 30/1027 (2.9%) | 31/994 (3.1%) | 37/1025 (3.6%) | 31/1003 (3.1%) |
| Door-to-needle <60 min | 33/1006 (3.3%) | 26/898 (2.9%) | 30/1027 (2.9%) | 31/994 (3.1%) | 37/1025 (3.6%) | 31/1003 (3.1%) |
| Direct stroke unit adm. | 75/11783 (0.6%) | 63/10586 (0.6%) | 60/11569 (0.5%) | 78/11363 (0.7%) | 82/11470 (0.7%) | 74/10756 (0.7%) |
| Swallowing test | 0/11783 (0.0%) | 0/10586 (0.0%) | 0/11569 (0.0%) | 0/11363 (0.0%) | 0/11470 (0.0%) | 0/10756 (0.0%) |
| Physiotherapy <48 h | 1365/11783 (11.6%) | 1225/10586 (11.6%) | 1337/11569 (11.6%) | 1340/11363 (11.8%) | 1293/11470 (11.3%) | 1220/10756 (11.3%) |
| Occupational therapy <48 h | 1434/11783 (12.2%) | 1277/10586 (12.1%) | 1362/11569 (11.8%) | 1398/11363 (12.3%) | 1371/11470 (12.0%) | 1277/10756 (11.9%) |
| 7-day survival | 2/11783 (0.0%) | 1/10586 (0.0%) | 1/11569 (0.0%) | 0/11363 (0.0%) | 1/11470 (0.0%) | 0/10756 (0.0%) |
| 90-day survival | 2/11783 (0.0%) | 1/10586 (0.0%) | 1/11569 (0.0%) | 0/11363 (0.0%) | 1/11470 (0.0%) | 0/10756 (0.0%) |

|  | July | August | September | October | November | December |
| --- | --- | --- | --- | --- | --- | --- |
| Thrombolysis | 11/9219 (0.1%) | 5/9503 (0.1%) | 9/9417 (0.1%) | 52/9732 (0.5%) | 35/9306 (0.4%) | 12/9614 (0.1%) |
| Door-to-needle <30 min | 44/954 (4.6%) | 34/1015 (3.3%) | 36/988 (3.6%) | 20/1007 (2.0%) | 22/1006 (2.2%) | 27/1096 (2.5%) |
| Door-to-needle <60 min | 44/954 (4.6%) | 34/1015 (3.3%) | 36/988 (3.6%) | 20/1007 (2.0%) | 22/1006 (2.2%) | 27/1096 (2.5%) |
| Direct stroke unit adm. | 85/10483 (0.8%) | 76/10905 (0.7%) | 60/10771 (0.6%) | 57/11179 (0.5%) | 69/10812 (0.6%) | 73/11067 (0.7%) |
| Swallowing test | 1/10482 (0.0%) | 0/10905 (0.0%) | 0/10771 (0.0%) | 0/11179 (0.0%) | 0/10812 (0.0%) | 0/11067 (0.0%) |
| Physiotherapy <48 h | 1185/10483 (11.3%) | 1284/10905 (11.8%) | 1247/10771 (11.6%) | 1307/11179 (11.7%) | 1261/10812 (11.7%) | 1296/11067 (11.7%) |
| Occupational therapy <48 h | 1208/10483 (11.5%) | 1277/10905 (11.7%) | 1229/10771 (11.4%) | 1320/11179 (11.8%) | 1263/10812 (11.7%) | 1295/11067 (11.7%) |
| 7-day survival | 1/10483 (0.0%) | 1/10905 (0.0%) | 1/10771 (0.0%) | 2/11179 (0.0%) | 1/10812 (0.0%) | 1/11607 (0.0%) |
| 90-day survival | 1/10483 (0.0%) | 1/10905 (0.0%) | 1/10771 (0.0%) | 2/11179 (0.0%) | 1/10812 (0.0%) | 1/11607 (0.0%) |
